# A Comparison of Three Neural Network Approaches for Estimating Joint Angles and Moments from Inertial Measurement Units

**DOI:** 10.3390/s21134535

**Published:** 2021-07-01

**Authors:** Marion Mundt, William R. Johnson, Wolfgang Potthast, Bernd Markert, Ajmal Mian, Jacqueline Alderson

**Affiliations:** 1 Minderoo Tech and Policy Lab, UWA Law School, The University of Western Australia, Crawley 6009, Australia; jacqueline.alderson@uwa.edu.au; 2 Houston Astros Baseball Club, Houston, TX 77001, USA; bill@johnsonwr.com; 3 Institute of Biomechanics and Orthopeadics, German Sport University Cologne, 50933 Cologne, Germany; potthast@dshs-koeln.de; 4 Institute of General Mechanics, RWTH Aachen University, 52062 Aachen, Germany; markert@iam.rwth-aachen.de; 5 School of Computer Science and Software Engineering, The University of Western Australia, Crawley 6009, Australia; ajmal.mian@uwa.edu.au; 6 Sports Performance Research Institute New Zealand (SPRINZ), Auckland University of Technology, Auckland 1010, New Zealand

**Keywords:** machine learning, wearable sensors, joint kinematics, joint kinetics

## Abstract

The application of artificial intelligence techniques to wearable sensor data may facilitate accurate analysis outside of controlled laboratory settings—the holy grail for gait clinicians and sports scientists looking to bridge the lab to field divide. Using these techniques, parameters that are difficult to directly measure in-the-wild, may be predicted using surrogate lower resolution inputs. One example is the prediction of joint kinematics and kinetics based on inputs from inertial measurement unit (IMU) sensors. Despite increased research, there is a paucity of information examining the most suitable artificial neural network (ANN) for predicting gait kinematics and kinetics from IMUs. This paper compares the performance of three commonly employed ANNs used to predict gait kinematics and kinetics: multilayer perceptron (MLP); long short-term memory (LSTM); and convolutional neural networks (CNN). Overall high correlations between ground truth and predicted kinematic and kinetic data were found across all investigated ANNs. However, the optimal ANN should be based on the prediction task and the intended use-case application. For the prediction of joint angles, CNNs appear favourable, however these ANNs do not show an advantage over an MLP network for the prediction of joint moments. If real-time joint angle and joint moment prediction is desirable an LSTM network should be utilised.

## 1. Introduction

Inertial measurement unit (IMU) sensors are gaining traction as a clinical gait analysis tool due to their improved accuracy, feasibility, ease-of-use, and importantly, applicability outside of the laboratory environment [1,2]. A meta-analysis of inertial sensor based gait analysis research concluded limited evidence in their application for determining joint kinematics, especially in non-sagittal (flexion-extension) motion [3]. Traditional biomechanical modelling methods developed using gold-standard three-dimensional (3D) motion capture marker data require the user to establish an underlying anatomical model [4]. To do this using IMU sensors, it is necessary to determine the sensor orientation with respect to the segment orientation in a global reference system (sensor-to-segment alignment). A variety of methods can be adopted for this purpose, all of which are dependent on sensor fusion algorithms and determining the initial sensor-to-segment alignment via the implementation of calibration postures or functional movements that the participant must execute [5]. Acceptable levels of within- and between-participant, and within- and between-tester repeatability, can only be achieved with appropriate sensor fusion algorithm application and accurate and reliable calibration procedures [6,7,8]. A limited number of papers have attempted to directly estimate joint moments and ground reaction forces (GRF) using inertial sensors, with efforts to predict GRFs limited in accuracy and constrained to predictions of vertical component or peak values only. The direct determination of GRFs based on Newton’s equations is challenging as their solution is indeterminate during the double support phase. The sensors provide the acceleration of segments or the body’s centre of mass (COM) (if they are attached close to the COM) which can be used as a surrogate measure of force during the single support phase, however more advanced algorithms are required for the determination of the GRF during the double support phase. A variety of methods have been proposed for this purpose, and reviewed by Ancillao et al. [9]. To overcome accuracy limitations and the restricted subsets of parameters that can be determined, researchers have focused on applying machine learning methods to improve the prediction of GRFs, joint angles and joint moments [2,10,11,12,13,14,15], with initial efforts focused on predicting smaller subsets of data, such as single GRF and joint moment components [10,11,12], or in the case of Stetter et al. [13] by predicting sagittal and frontal plane moments in isolation. Very recently gait researchers have trained machine learning models to predict all component joint angles [14,15] and moments across all lower limb joints [14].

ANNs learn connections between given (IMU data) input and target data (e.g., joint kinematics/kinetics). For training purposes, datasets need to contain IMU data (inputs) and 3D marker motion capture data (targets), so the target data for the ANN model is anatomically standardised, a requirement for gold-standard biomechanical models [4]. Subsequently, the trained model can be used to predict the target parameters, hence only inertial sensors are required. On the downside, ANN models can only predict relationships they have learned during the training process. This means, if an ANN has been trained on IMU data collected with a specified sensor-to-segment alignment, it will only predict accurate joint kinematics/kinetics when the alignment in new data is replicated. To overcome this, a large variance in IMU sensor-to-segment alignment in the training dataset is necessary. In addition to the requirement of an adequate dataset, the choice of ANN model is important. Three different classes (and hybrids) of ANNs have been been used in biomechanics with each having advantages and disadvantages as summarily outlined below. For a more detailed explanation of ANNs please refer to Goodfellow et al. [16].

- Multilayer perceptron (MLP) networks are the simplest and classical class of ANNs. They are flexible and are used to learn relationships between inputs and outputs for classification or regression tasks. By flattening image or time series data, MLP networks can be used to process time-sequence input data. They are easy to train but are limited to time-normalised data and are computationally expensive. They provide baseline information for the predictability of all types of data. IMU sensor data has been used as input into this type of ANN for the prediction of joint angles and joint moments [12,13,14,17].
- Convolutional Neural Networks (CNNs) were originally designed to map image data to a single output variable, a classification task. They learn from raw image data by exploiting correlations between local pixels. They work especially well on data with a spatial relationship, and due to the fact an ordered relationship can also be found in time series data, this makes CNNs suitable for time series prediction of human motion. CNNs have been used with inertial sensor data inputs to predict joint kinematics and kinetics [11,15]. Different open-access models have been trained on large datasets for image classification previously, enabling the use of transfer learning or fine tuning of a model instead of training a CNN from scratch. To be able to use models trained on large image databases and apply transfer learning rather than training from scratch, Johnson et al. [18,19] transformed motion time sequences to images for the prediction of three dimensional knee joint moments and ground reaction force sequences based on motion capture data inputs.
- Recurrent Neural Networks, such as Long Short-Term Memory (LSTM) networks, were designed for sequence prediction problems. They make use of time dependencies in data which explains their success in natural language processing. Hence, they are also convenient for tasks involving the prediction of motion sequences. Unfortunately, LSTM networks are intensive to train and require large datasets, something rarely available in biomechanics [2]. Using inertial sensor data as inputs, LSTM networks have also been previously used to predict joint angles and moments [15,17,20].

No previous study has recommended which class of ANN is optimal for the prediction of biomechanical time series data. This paper aims to compare the prediction accuracy of three ANNs by applying the proposed technique to the same source dataset. For this purpose, inertial sensor data—acceleration and angular rate—is simulated based on marker trajectories captured using a 3D retro-reflective camera-based motion analysis set-up. This data is used to leverage a sparse dataset of level walking trials using inertial sensors. Three dimensional angles and moments of the hip, knee and ankle joint are predicted based on the inertial sensor data from the pelvis, and bilateral thighs and shanks. An MLP, LSTM and CNN is trained for the prediction task. Since a rather large dataset is available, the CNN is trained from scratch and a pretrained CNN is used. Based on the results of previous research, we expect the (pretrained) CNN to outperform the MLP and LSTM neural networks.

## 2. Materials and Methods

### 2.1. Dataset

The dataset comprised aggregated level walking data, sourced from a series of independent studies [14,21,22,23]. All data was sourced by the German Sport University Cologne and was approved by the University Ethics Committee (approval no. 025/2014, 010/2017, 154/2018, 133/2019) with all participants providing informed written consent. The dataset comprised 116 participants (48 female, 37.6 ± 17.1 years, 72.5 ± 11.9 kg, 1.73 ± 0.09 m). Each participant executed level walking trials at self-selected speeds ranging from 0.8 to 2.0 m/s. All participants’ motion was recorded using an opto-electronic motion capture system (VICON^®^, MX F40, Oxford, UK, 100–125 Hz) and two force plates (Kistler Instrumente AG, Winterthur, Switzerland, 1000 Hz). Twenty-eight retro-reflective markers were attached to defined bony landmarks of the lower body to create a rigid body model [24]. Concurrent with the optical motion capture, 23 of the 116 participants were additionally fitted with five custom inertial sensors to record the linear acceleration and angular rate of the pelvis, and bilateral thigh and shank (100 Hz, TinyCircuits, Akron, OH, USA). An Android based custom application collected the IMU data on a smartphone [14,23].

### 2.2. Data Processing

The optical motion capture data wee filtered using a zero-lag fourth-order low-pass Butterworth filter with a cut-off frequency of 6 Hz. The 3D joint moments of the hip, knee, and ankle joint were calculated using the AnyBody^®^ Modeling System (Version 6.0, AnyBody Technology, Aalborg, Denmark) and normalised to each participant’s body weight and height. The joint angles were calculated using a custom MATLAB script based on the recommendations of the International Society of Biomechanics [4,24,25]. IMU acceleration and angular rate was filtered using a zero-lag first-order low-pass Butterworth filter with a cut-off frequency of 5 Hz. Both inertial and motion capture data were synchronised using an optimisation approach [14].

Inertial sensor data were simulated based on marker trajectories placed on the pelvis, thighs and shanks in a multi-step process. In step one, the segment coordinate systems of the hip, knee and ankle joints are determined [4] as quaternions. These coordinate systems are translated and rotated in the subsequent step to match sensor positions and initial orientations used during the data collection using an optimisation approach. The dataset used in this study contains ground-truth inertial sensor data from 23 participants. To ensure a wide variability in the range of synthesised IMU data, the initial position and orientation were optimised to match each participant’s ground truth sensor position and orientation. Thus, all 23 slightly different positions and orientations of the ground truth data were simulated for each participant in the motion capture dataset. This resulted in data augmentation by a factor of 23. In a final step, the quaternion derivative was calculated to determine the angular rate, and the second derivative of the simulated sensor’s origin was used to calculate the acceleration of the simulated sensor. For a more detailed description of the simulation refer to Mundt et al. [14]. The simulated data comprised 3D acceleration and 3D angular rate of five sensors—an input matrix containing 30 time series features. The output matrix (joint angles or joint moments of the bilateral hip, knee and ankle joint) contained 18 time series features. All data was time normalised to stance (kinetics) or step (kinematics) phases resulting in time series of 101 data points.

The multiple ANNs required different data input configurations. To apply an MLP, the motion sequences needed to be flattened as this type of ANN cannot model time dependencies. During the flattening process the third dimension (time) was removed from the data by concatenating the time series. This results in a two-dimensional input and output matrix of size [number of samples × number of features] with numbers of features being (number of input/output features × time steps). As an LSTM learns time dependencies, this dimension needs to be maintained in the input and output data which results in a three-dimensional input and output matrix of size [number of samples × number of input/output features × time steps]. Conversely, CNNs are primarily used for classifying image data. To be adopted for our purpose, the motion sequences were transformed to RGB images with each channel displaying one direction of the sensor signals. The resulting matrix is interpolated to match the size of previously used images. The four dimensional input matrix is of size [number of samples × 224 × 224 × 3] and the two-dimensional output matrix of size [number of samples × (number of output features × time steps)]. For further information on the transformation of motion sequences to images please refer to Johnson et al. [18,19].

A sanity check was performed on all input and output features to avoid low prediction accuracy attributed to low data quality. The simulation process and the optimisation approach to synchronise the time sequences might result in discrepancies in the data. These sequences were excluded from the dataset by only using those samples within a 95% confidence interval.

### 2.3. Neural Network Application

**Figure 1:**
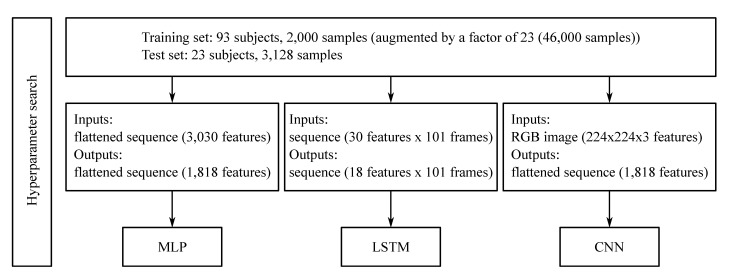
Exemplar overview of the work flow for the determination of the kinematics hyperparameters. First, the dataset consisting of 3D acceleration and 3D angular rate of five inertial sensors, and bilateral hip, knee and ankle joint 3D joint angles is split into training and test sets. Different preprocessing steps are then undertaken to create appropriate input and output data shapes for the training and testing of the three neural networks: MLP, LSTM and CNN.

The process of training the ANN was divided into two steps: in the first, the best hyperparameters for the models were determined using simulated inertial sensor data (i.e., synthesised IMU data) from the participants who had 3D motion capture data but did not have recorded (ground-truth) inertial sensor data. The dataset was split into a training and validation set while the ground truth IMU data was used as the test set. To determine the best hyperparameters for the multilayer perceptron (MLP), the long short-term memory (LSTM) and the final two dense layers of the convolutional neural network (CNN) an automated hyperband search [20,26] was executed (Figure 1).

**Figure 2:**
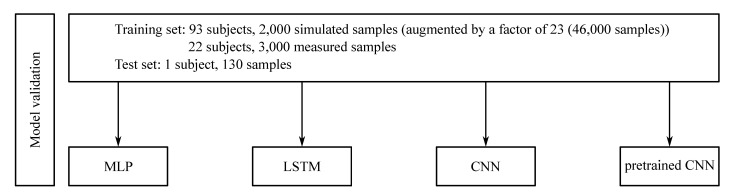
Overview of the work flow for the validation of the trained models. A leave-one-subject-out (LOSO) validation exemplar is displayed for the kinematics and was performed using the optimised hyperparameters for the four different models: MLP, LSTM, CNN and pretrained CNN. Based on this validation the model performance was assessed.

In the second step, a leave-one-subject-out (LOSO) validation was performed. All simulated IMU data plus all ground-truth IMU data, excluding the trials of one participant, were used to train the ANN using the optimised hyperparameter set. Hence, no validation set was necessary. The trials from the participant left out in the training process were used then as test set (Figure 2).

**Figure 3:**
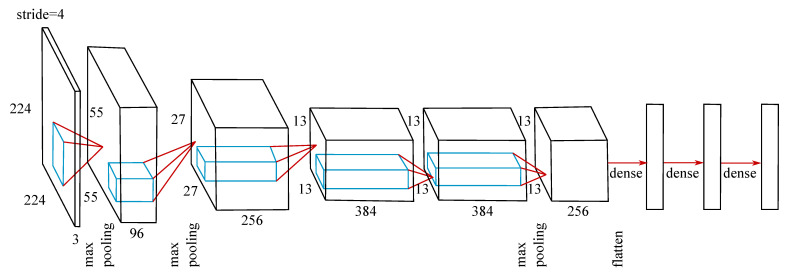
Architecture of the applied convolutional neural network. The size of the dense layers was found during the hyperband search and differs for the joint angle and joint moment prediction. For the pretrained model, the convolutional and pooling layers were set to be non-trainable.

The MLP and LSTM were trained from scratch. The CNN was trained from scratch and transfer learning was applied. For this purpose, the weights of the convolutional layers of a publicly available pretrained caffenet mode [https://github.com/BVLC/caffe/tree/master/models/bvlc_reference_caffenet, accessed on 1 October 2020] were applied (Figure 3).

The caffenet model files were transformed to be compatible with the Tensorflow framework used throughout this study [27]. To use the pretrained weights, the convolutional and pooling layers of the ANN were defined as non-trainable, hence only the dense layers’ weights were adapted during the training process.

To compare the predictions of the different ANNs, the root-mean-square error normalised to the range of the data (nRMSE) and the correlation coefficient were analysed. The evaluation of the nRMSE rather than the root-mean-square error allows for the comparison between kinematic and kinetic predictions, in addition to direct comparisons between different motion planes. As walking gait comprises small ranges of motion in the non-sagittal motion planes, small deviations in joint angle prediction can lead to large nRMSE values. Joint moments were normalised to body weight and height and consequently between motion plane differences were less pronounced. The correlation coefficient does not take differences in range into consideration but cannot account for offsets between predicted and measured time series. Therefore, correlation coefficients and nRMSE values are presented to provide a comprehensive overview of model performance. The results are presented for all data within a 95% confidence interval.

## 3. Results

The optimal results for the architecture and hyperparameter search for all models are presented in Table 1. It was ensured that none of the found parameters equal the upper or lower boundaries of the search.

**Figure 4:**
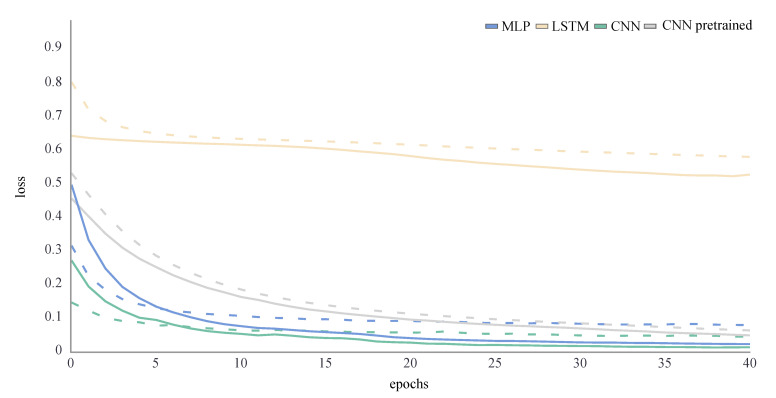
Loss curve of the joint angle training process of an exemplary LOSO validation for all ANNs. The training loss is displayed by a dashed line, while the validation loss is displayed by a solid line.

**Figure 5:**
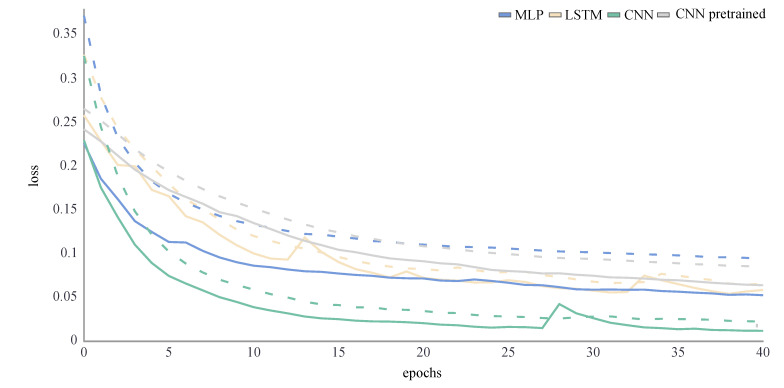
Loss curve of the joint moment training process of an exemplary LOSO validation for all ANNs. The training loss is displayed by a dashed line, while the validation loss is displayed by a solid line.

Using these architectures and hyperparameters, all ANNs were trained for 40 epochs. The training and validation loss were a performance measure for ANNs and described the mean absolute error between the ground truth and predicted values for each epoch of the training process. The training loss decreased throughout the whole training process. If the model perfectly learned the features in the training set, it would tend towards zero. The validation loss was an indicator for overfitting: it increased at some point during the training, when the ANN started learning features that were specific in the training data but not in the validation data. To find the optimum number of training epochs both training and validation loss needed to be considered [16]. To avoid overfitting and a reduced generalisability of the model, the training was stopped as soon as the validation loss did not decrease any further. No overfitting was observed during the training process for any ANN (Figure 4 and Figure 5). For the joint kinematics, the final loss of the LSTM network was distinctly higher than for the other ANN. The pretrained CNN performed slightly worse than the MLP and CNN, which returned similar loss values. For the joint kinetics, the final loss was very similar for the MLP, LSTM and pretrained CNN, while the CNN loss was even smaller.

**Figure 6:**
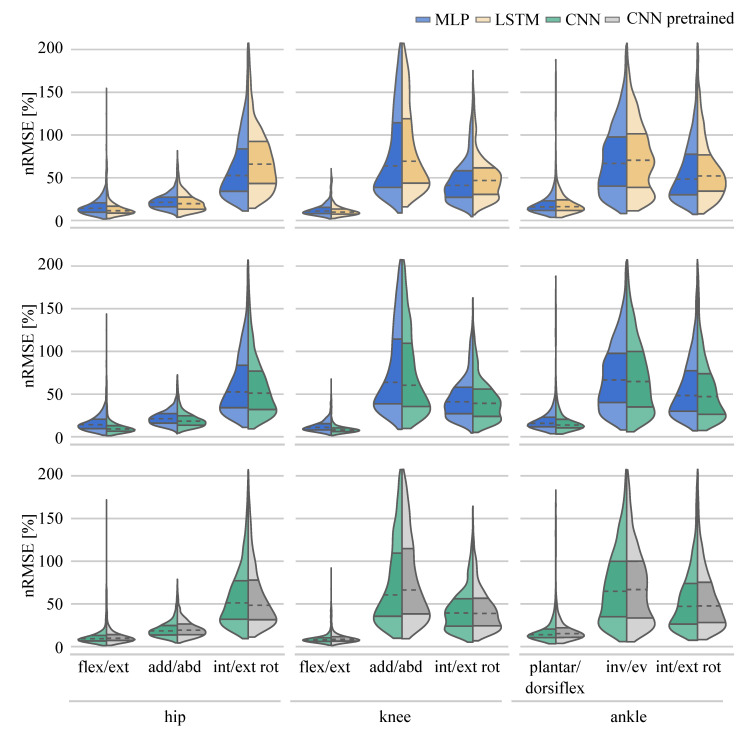
Distribution of the nRMSE values for the joint angle prediction.

**Figure 7:**
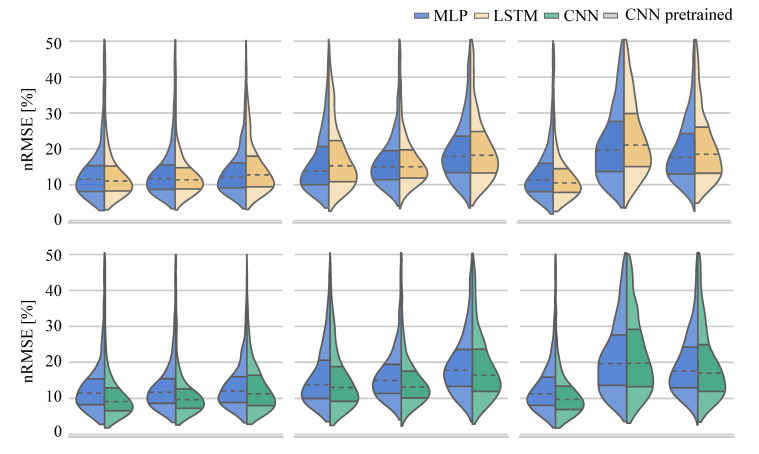

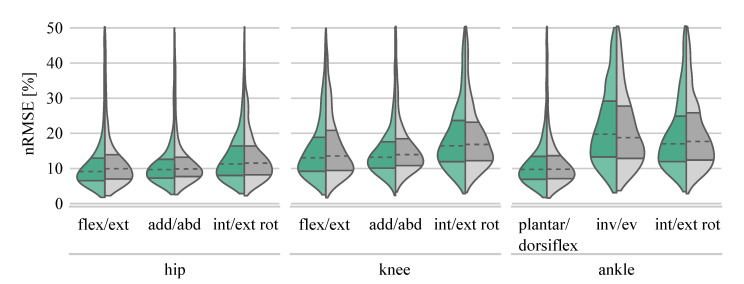
Distribution of the joint moment prediction RMSE values.

To compare the performance of the different ANNs on the same task, the distribution of the nRMSE is displayed in joint kinematic (Figure 6) and joint kinetic (Figure 7) violin plots. While the distribution of the nRMSE was similar for the kinetics prediction, differences between the LSTM and MLP model violin shapes were observed for the kinematic prediction. This was to be expected given the higher final loss value reported for the LSTM.

The MLP, as the simplest ANN, was considered the baseline network for performance comparisons. Compared with the MLP, the LSTM performed worse overall for the prediction of joint angles (−2.70%). The LSTM performed better than the MLP in predicting hip flexion-extension and adduction-abduction angles, and knee flexion-extension angles. However, prediction performance was worse for hip internal-external rotation, knee adduction-abduction and internal-external rotation angles, and for all ankle joint angles. A CNN trained from-scratch model resulted in a consistent overall improvement in prediction (11.89%) across all lower limb joint angles (hip, knee and ankle). A pretrained CNN resulted in an overall worse performance when compared to the prediction performance of the CNN trained from scratch (−3.76%). Only hip and knee internal-external rotation angle predictions were improved. All other pretrained CNN joint angle predictions were poorer than the CNN trained from scratch (Table 2, Figure 6).

When considering joint kinetic prediction ANN performance, overall the LSTM performed slightly worse (−1.73%) than the MLP network. On closer examination, the LSTM outperformed the MLP on the prediction of hip flexion-extension and adduction-abduction moments, knee adduction-abduction moments and ankle plantar-dorsiflexion moments. The CNN overall performed nearly ten percent better than the MLP (9.63%). Only for the prediction of ankle inversion-eversion moments the CNN did not outperform the MLP. Overall, using a pretrained CNN did not result in a better joint moment prediction performance than a CNN trained from scratch (−2.80%), with only ankle inversion-eversion moments using a pretrained CNN reporting superior prediction results (Table 2, Figure 7).

**Figure 8:**
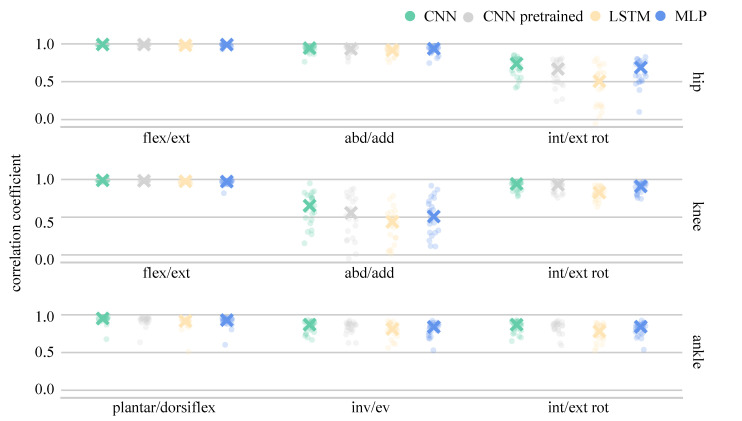
Median and single LOSO validation correlation coefficients for the prediction of joint angles.

**Figure 9:**
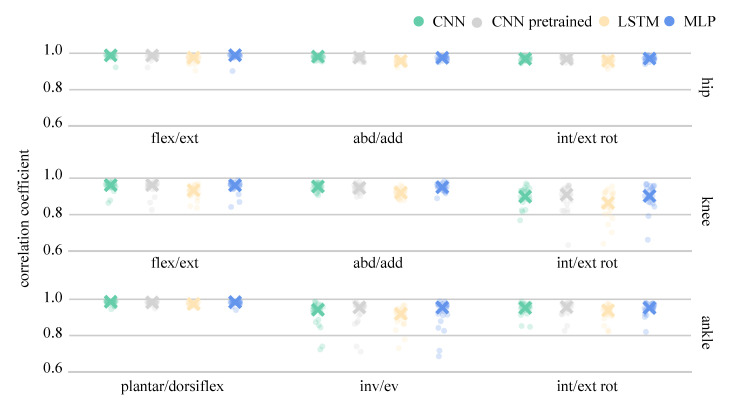
Median and single LOSO validation results of the correlation coefficient for the prediction of joint moments.

The analysis of single LOSO validations, as well as the median correlation coefficients for all joint angles are presented in Figure 8 and for joint moments in Figure 9. Joint angle prediction was more difficult than joint moment prediction for all the ANNs with mean model correlation coefficients ranging between 0.788 and 0.874. Although flexion-extension angles were predicted with high correlations (>0.911), knee adduction-abduction angles presented a major challenge for all ANNs, resulting in correlation coefficients ranging between 0.389 to 0.625. A huge variance was found between the single LOSO validation runs and, generally, all ANNs showed similar variance pattern trends. The trained from-scratch CNN outperformed all other ANNs, the pretrained CNN and MLP performed equally well, while the LSTM achieved the lowest correlation coefficient. The average combined model joint moment correlations exceeded 0.939. Only knee internal-external rotation and ankle inversion-eversion moments returned correlations below 0.8 for single LOSO validations, a trend observed across all ANN prediction results. Generally, the performance of the from-scratch CNN, the pretrained CNN and the MLP were relatively similar, while the LSTM was the worst performing ANN when LOSO validation was applied.

## 4. Discussion

This paper aimed to provide insight into the performance of different ANNs when applied to the task of predicting joint angles and joint moments based on acceleration and angular rate signals of the lower limbs. The dataset used was augmented by simulated inertial data based on gold-standard optical motion capture 3D data. The adoption of this approach meant that the simulated data enhanced the generalisability of the training set to suitably accommodate all of the measured IMU data. This resulted in a training dataset of close to 50,000 samples of more than 100 participants. The prediction was evaluated using directly recorded inertial sensor data–acceleration and angular rate–using a LOSO cross-validation. In previous studies it has been shown that predictions from models trained and tested using simulated data overestimate prediction accuracy [17], but simulated data can be used to support predictions based on measured data [14].

In contrast to previous work by the current authors [14,17], the model architecture and hyperparameter search was automated for improved time-efficiency. This resulted in the identification of different architectures and hyperparameters than adopted previously using a manual approach analysing the same task and dataset [14]. Previously published joint angle prediction correlations were slightly higher (0.85 [14] vs. 0.832 current) and joint moment prediction lower (0.95 [14] vs. 0.962 current) than those observed in the present work. Mundt et al. [17,28] previously published LSTM networks that used simulated inertial sensor data or joint angle data as inputs to predict joint moments. This previously published data returned lower correlations than the comparator MLP network, a finding consistent with the results of the present study. However, LSTM neural networks excel in dealing with data comprising arbitrary sequence lengths. In order to ensure the tested ANNs received the exactly same data in this study, input data was required to be time-normalised, despite it being previously shown that this leads to an underestimation of the prediction capabilities of an LSTM network [20].

Johnson et al. [29] applied transfer-learning to the same CNN used in this study, [https://github.com/BVLC/caffe/tree/master/models/bvlc_reference_caffenet, accessed on 1 October 2020] to predict the three dimensional knee joint moments based on 3D retro-reflective marker trajectories. When compared with the results from the present study, they reported very high correlations for knee flexion-extension and adduction-abduction joint moments (0.98 [29] vs. 0.96 current, 0.96 [29] vs. 0.95 current) but a lower correlation for knee internal-external rotation (0.68 [29] vs. 0.91 current). The CNN model in the present study was trained from scratch and transfer-learning was used. During this process the weights of the convolutional part of the ANN trained for an image classification task were used, while the final dense layers were replaced by trainable layers of the optimum size found during the automated search. The CNN trained from scratch joint moment correlation coefficients were similar to the pretrained CNN and the MLP network. This indicates that the final dense layers of the model are more important for the prediction task than the convolutional structures. For the prediction of joint angles, the CNN trained from scratch outperformed the pretrained CNN, and both CNNs performed better than the MLP. Contrary to the joint moment prediction this may show that the convolutional layers are able to extract the relevant features from the input images, allowing for overall better performance. One possible explanation for this result is the variability in the initial start values of the joint angles. While joint moments time-normalised to the stance phase will always display an initial value very close to zero, time normalised joint angle data will have varying initial values at foot contact–the commencement of the stance phase. This behaviour cannot be captured well by MLP networks (and LSTM networks), hence the CNN’s strength in feature extraction is likely exploited to overcome this problem.

The LOSO validation correlation coefficients show high variability. This variability was consistently observed in all tested ANNs. Possible explanations include that specific participants show a movement pattern that substantially digresses from the majority of other participants, and consequently this motion was not well covered by the training set. A second explanation may be that specific participant data is of lower quality. To mitigate against this an accuracy risk attributed to low quality input data, an automated data sanity check was performed during preprocessing and only those steps within a 95% confidence interval were evaluated. This sanity check did exclude faulty sequences but may have also served to reduce the variability in the dataset.

All ANNs evaluated in this study show very good overall results for the prediction of both joint kinematics and kinetics. These results are encouraging given the prediction of joint kinetics is of high relevance to gait practitioners and those looking to advance sports biomechanics outside of the laboratory. Especially given the difficulty in predicting joint kinetics, which are prone to significant error when directly estimated from inertial sensor data in isolation (i.e., without using advanced analytics or machine learning techniques). This study provides an overview of the applicability, advantages, and disadvantages, of three commonly used neural networks, but cannot deliver a distinct recommendation of which network to use for different datasets or applications. In future work, the applicability of different ANNs on datasets of different tasks and different sizes should be analysed. Additionally, the influence of filtering techniques on the inertial data should be evaluated.

## 5. Conclusions

For the dataset presented in this study the application of a CNN trained from scratch returned the most favourable predictions. However, this requires a large dataset and elaborate preprocessing steps to convert inertial sensor data to images. An MLP shows similar or only slightly worse prediction capabilities, with significantly fewer preprocessing stages. The performance of an LSTM was inferior but demands even less preprocessing and facilitates near real-time application. This was the first study that applied three different types of commonly used neural networks to the same dataset. The results showed that a high prediction accuracy could be achieved with all three neural networks suggesting that the dataset itself and the prediction task is more relevant than the neural network used.

## Figures and Tables

**Table 1:** Neural Network architectures and hyperparameters.

Joint Angles				
ANN	Layers	Learning Rate	Dropout	Activation
MLP	6000–4000	0.0003	0.5	relu
LSTM	32–32	0.0003	0.7	tanh
CNN	3000–6000	0.00003	0.4	relu
**Joint Moments**				
**ANN**	**Layers**	**Learning Rate**	**Dropout**	**Activation**
MLP	3000–1000	0.0003	0.5	relu
LSTM	128–1024	0.0003	0.4	tanh
CNN	2000–4000	0.0001	0.4	relu

**Table 2:** Comparison of the different neural networks. Values display the difference in nRMSE values. Positive values show an improvement compared to the baseline (MLP or CNN from scratch).

		Joint Angles		
		**LSTM vs. MLP [%]**	**CNN vs. MLP [%]**	**Pretrained CNN vs. CNN [%]**
	flex/ext	20.25	35.57	−5.79
hip	add/abd	7.47	13.43	−4.81
	int/ext rot	−25.20	2.80	5.40
	flex/ext	11.41	27.70	−6.38
knee	add/abd	−8.55	5.31	−9.50
	int/ext rot	−14.03	4.18	1.21
	plantar−dorsiflex	−2.38	12.53	−9.88
ankle	inv/ev	−5.61	2.87	−3.03
	int/ext rot	−7.67	2.61	−1.08
		**Joint Moments**		
		**LSTM vs. MLP [%]**	**CNN vs. MLP [%]**	**Pretrained CNN vs. CNN [%]**
	flex/ext	4.13	20.64	−9.27
hip	add/abd	3.07	17.29	−1.83
	int/ext rot	−5.73	6.37	−2.07
	flex/ext	−10.49	5.59	−4.01
knee	add/abd	0.18	12.17	−5.76
	int/ext rot	−1.81	7.89	−2.39
	plantar−dorsiflex	7.30	13.90	−0.83
ankle	inv/ev	−7.39	−0.62	4.92
	int/ext rot	−4.79	3.37	−3.99

## Data Availability

The data presented in this study are available on request from the corresponding author. The data are not publicly available due to source data participant consent limitations.
